# An Integrative Review Exploring Womens’ Experiences of Retraumatization Within Perinatal Services

**DOI:** 10.1111/jmwh.13662

**Published:** 2024-07-22

**Authors:** Jennifer Gordon, Andrew Hunter, Fiona Callanan, Clare Kiely, Annmarie Grealish

**Affiliations:** ^1^ Department of Nursing and Midwifery University of Limerick Limerick Ireland; ^2^ School of Nursing and Midwifery University of Galway Galway Ireland; ^3^ University Maternity Hospital Limerick Limerick Ireland; ^4^ Florence Nightingale Faculty of Nursing Midwifery & Palliative Care, King's College London London United Kingdom

**Keywords:** integrative review, maternity services, perinatal mental health, re‐traumatization, trauma, trauma‐informed care

## Abstract

**Introduction:**

Evidence indicates that retraumatization has a detrimental effect for those women who are accessing perinatal services. One in five women worldwide has a history of childhood adversity. Between 18% and 34% of women experience trauma, which is a well‐known risk factor for the onset of chronic mental health disorders. There is a lack of evidence on women's experiences on retraumatization in perinatal care settings and how to prevent retraumatization from occurring. The purpose of this study was to conduct an integrative review on women experiences of retraumatization to determine preventive measures within perinatal services.

**Methods:**

This integrative review followed Whittemore and Knafl's 5‐stage framework as it allows for the inclusion and integration of diverse research methodologies into an overall synthesis of the evidence. A systematic search of 5 databases was conducted (Web of Science, MEDLINE, CINAHL, ASSIA, and PsychINFO) with no date, language, or geographical limits set due to the paucity of research published in this subject area. This review was conducted and reported according to the Preferred Reporting Items for Systematic Reviews and Meta‐Analyses guidelines.

**Results:**

Fifteen studies met the inclusion criteria and were included in the thematic synthesis. The review identified that participants across the studies had a history of child sexual abuse, sexual abuse, and rape. Three main themes plus subthemes were identified: (1) activating (subthemes: positions in labor, intimate procedures, communications with health care professionals, loss of control); (2) outcomes (subtheme: emotional responses); and (3) interventions reducing or preventing retraumatization (subthemes: role of the health care professional, screening for abuse and history of trauma).

**Discussion:**

Our findings demonstrate that women are experiencing retraumatization in perinatal services, and there is evidence of formalized approaches being applied in clinical settings to prevent retraumatization from occurring. This study is the first to examine the factors that contribute to retraumatization in perinatal services and make recommendations to reduce the harmful practices in place in perinatal care settings.

## INTRODUCTION

Psychological health during pregnancy and the postpartum period is receiving growing attention, and the importance of this is globally recognized.^1^, ^2^ There is mounting evidence that the perinatal period, which extends from the onset of pregnancy to 12 months after childbirth,^3^ is the most vulnerable time in a woman's life.^4^, ^5^ This critical time marks substantial changes to women's physiological, social, and psychological health, leading to increased risks for negative physical health outcomes and mental health sequelae, including depression, anxiety, and posttraumatic stress disorder (PTSD).^4^, ^5^, ^6^, ^7^, ^8^, ^9^

1 **QUICK POINTS**

✦ Multiple studies have found associations between traumatic experience and adverse outcomes for women resulting in mental health consequences and risks for posttraumatic stress disorder.
✦ A framework to help perinatal health care staff prevent retraumatization does not exist.
✦ Evidence suggests that during the perinatal period, women are not screened for previous trauma histories on admission, and their trauma history is not taken into consideration during procedures.
✦ Positioning during labor, intimate examinations, communications with health care professionals, and loss of control can reactivate previous traumatic experiences for women.

The prevalence of trauma among people with mental disorders has been found to be at least 2 to 3 times higher than in the general population, and this recognition has led to an increase in research on women's experiences of trauma and risk for PTSD.^10^, ^11^, ^12^ Studies have found that a history of childhood maltreatment including physical, sexual, and emotional abuse and physical and emotional neglect can lead to mental health sequelae and adversely affect women's perinatal health, pregnancy outcomes, perinatal health risks, postpartum mental health, bonding, family relationships, and parenting.^13^, ^14^, ^15^ The impact of these physical and mental health consequences can also adversely affect short‐ and long‐term developmental trajectories for infants and children.^16^, ^17^

The World Health Organization^18^ has reported that 1 in 5 women worldwide has a history of adverse childhood experiences (ACEs). Numerous studies in the United States suggest that approximately 50% of children under 18 years of age have been exposed to at least one ACE event, with approximately 33% exposed to multiple ACEs and 21.5% experiencing 3 or more ACEs.^10^, ^12^, ^19^, ^20^ Likewise, 39% of female individuals in the US population^12^ have been exposed to multiple ACEs in their first 18 years of life and report a more complex and varied history of childhood adversities than male individuals.

Within the last decade, trauma and abuse, including physical trauma (physical trauma related to childbirth) and interpersonal trauma unrelated to pregnancy (trauma from childhood experiences, sexual assault, rape), have received growing attention. Multiple studies have found associations between traumatic experience and adverse outcomes for women resulting in mental health consequences and risks for PTSD.^4^, ^5^, ^7^, ^12^, ^21^, ^22^, ^23^, ^24^, ^25^, ^26^ The term *trauma* has been broadly defined and in this review is referred to as an event or circumstance that was experienced as harmful or life‐threatening with lasting impacts on mental, physical, emotional, or social well‐being.^25^, ^27^ Retraumatization is defined as an event a person experiences that reminds them of and activates an emotional response to a traumatic event.^19^, ^27^, ^28^ Retraumatization can cause the same reaction as the initial traumatic event.

A great deal has been learned about trauma and PTSD in women in the perinatal period, including factors that increase or decrease risks for PTSD, mental health symptoms, and comorbid conditions. To a lesser extent however, trauma exposure, abuse, and retraumatization are often not recognized in perinatal care settings.^29^ Women in contact with perinatal care services who have experienced childhood trauma may activate a relapse of preexisting mental health difficulties or symptoms related to past trauma. Reactivation can increase levels of anxiety and PTSD symptoms and is more likely to result in misuse of substance and alcohol, self‐harm, and attempted suicide.^1^, ^26^, ^29^, ^30^, ^31^ This, coupled with evidence of maternal mortality (which accounts for approximately 20% of postpartum deaths in the first year after a pregnancy), has led to the provision of perinatal mental health services to women and the development of trauma‐informed approaches across perinatal services.^19^, ^27^, ^28^, ^32^, ^33^

### Trauma‐Informed Care

Trauma‐informed care (TIC) aims to promote feelings of psychological safety, choice, and control. Its 6 core principles^27^ aim to improve care for all patients and include safety; trustworthiness and transparency; collaboration and mutuality; peer support, empowerment, voice, and choice; and cultural sensitivity. TIC helps health care professionals (HCPs) to recognize the signs, symptoms, and impact of trauma. This is achieved by integrating knowledge of trauma in a comprehensive way in service delivery, including perinatal mental health care.^1^, ^19^, ^28^, ^32^ TIC is now incorporated into the National Health Service,^34^ Health Service Executive,^35^ US Department of Health and Human Services,^1^ Substance Misuse and Mental Health Services Administration (SAMHSA),^27^ and Mental Health Coordinating Council.^36^ Because interpersonal trauma and retraumatization can strongly impact women's mental illness presentations and treatment trajectories, trauma exposure and abuse are often not recognized in perinatal care settings.^28^, ^29^ Evidence has suggested that perinatal staff may miss the connection between a woman's trauma history and the presenting problem, or the topic is avoided altogether due to a lack of knowledge and competence in providing TIC.^19^, ^37^, ^38^

Successive studies have shown that women accessing perinatal services have experienced trauma and retraumatization.^30^, ^31^, ^39^, ^40^, ^41^, ^42^ Research suggests that between 18% and 34% of women found their births traumatic, particularly women with history of ACE (specifically child sexual abuse [CSA]), and were more likely to suffer from retraumatization after being introduced into the perinatal services.^31^, ^43^, ^44^, ^45^ Kitzinger^45^ reported that a survivor described childbirth as “being sexually abused all over again.” Studies on survivors of sexual violence found that feelings of powerlessness, lack of control, lack of personal choice or autonomy, fear of physical examinations of any kind, or feelings of disgust toward themselves often confounded their experience of a traumatic birth.^43^, ^46^, ^47^

Given the adverse outcomes associated with trauma exposure, it is critical that midwives and other health care providers assess and screen women for history of trauma to improve recognition, provide postdisclosure support, and avoid the potential for retraumatization.^27^, ^39^, ^43^, ^46^, ^47^, ^48^ Koster et al^39^ found that the lack of information and lack of obtaining the woman's consent throughout the birthing experience resulted in feelings of being excluded and objectified. Therefore, it is vital that HCPs are aware of the trauma women may be subjected to or reliving during perinatal care and are aware of the signs of PTSD during the perinatal period. Due to the prevalence of trauma and the impact of trauma on health, we have an important opportunity to improve care for this vulnerable population of during the perinatal period.^28^, ^46^, ^47^

Although there are guidelines within perinatal care regarding the assessment of women's well‐being in the face of traumatic perinatal experiences, a framework to help perinatal health care staff prevent retraumatization does not exist. This represents a gap in the literature, indicating a need for consolidation of qualitative and quantitative evidence of women's experiences on retraumatization in perinatal care settings. Thus, this integrative review aims to synthesize the evidence to better understand women's experiences of retraumatization within perinatal services.

The authors acknowledge that not all pregnancy capable people identity as female. The terms *woman* and *women* will be used in this study to be consistent with the terminology from the included studies.

## METHODS

An integrative review^49^ was chosen to allow for the inclusion of diverse research methodologies into an overall synthesis of the evidence on women's experiences of retraumatization within perinatal services. Russell's^50^ 5‐stage integrative review process guided the methodological rigor of this review: (1) problem formulation, (2) literature search, (3) data evaluation, (4) data analysis, and (5) interpretation and presentation of the data. The protocol for this integrative review is reported in accordance with the Preferred Reporting Items for Systematic Reviews and Meta‐Analyses statement^51^ and was conducted at the same level of rigor as a systematic review using Cochrane guidance.^52^ The review protocol was registered on PROSPERO (CRD42022384640).

### Search Strategy

A preliminary search of publications outside of scientific journals (grey literature) was conducted within Proquest, Cochrane library, Open Grey database, and PROSPERO to ensure no other similar reviews had been conducted. A population, concept, context (PCC) framework analysis was next undertaken to guide the choice of keywords in the search strategy to formulate the following review question: *What are women's experiences of retraumatization within maternal services?*

Systematic searches of 5 electronic databases were conducted from inception to November 2022—Web of Science, MEDLINE, CINAHL, ASSIA, and PsychINFO—with no date, language, or geographical limiters. Identical searches were used for all databases and medical subject headings, and free‐text terms were used to guide the search (Supporting Information: Appendix S1). Boolean operators *OR* and *AND* were also used to refine and expand searches within and across groups of PCC terms. 

### Inclusion Criteria

Studies were included if the primary research (1) aimed at the experiences of trauma and retraumatization of pregnant women who were accessing or had accessed perinatal services; (2) focused on procedures and interventions that pregnant women found traumatizing or retraumatizing when accessing or had accessed maternity services; (3) were qualitative, quantitative, mixed‐methods design, systematic reviews, or case studies or series; and (4) sampled participants 18 years of age or older with self‐reported or diagnosed with either type 1 or type 2 trauma. Type 1 trauma is a single or one‐off event, and type 2 trauma or complex trauma occurs over an extended period of time, signifying trauma that is anticipated, repeated, or protracted.^53^ Studies were excluded if participants did not report a history of trauma or retraumatization when perinatal services were accessed or the data was not a result of a primary study (ie, protocols, abstracts, nonpublished theses, conferences, or non–peer reviewed journals).

### Study Selection

All data citations retrieved through the search were extracted and exported into the Covidence^54^ for de‐duplication and title, abstract, and full‐text screening. All titles and abstracts were independently reviewed by J.G. and A.G. to ensure the robust application of the inclusion and exclusion criteria. Full‐text articles of potential studies and papers retrieved through handsearching were then assessed for eligibility by J.G. and A.G. Discrepancies between reviewers were resolved collaboratively following title and abstract and full‐text screening with the advice of the third reviewer (A.H.).

### Quality Appraisal

Quality appraisal of included studies was assessed using the Joanna Briggs Institute (JBI) Critical Appraisal tools^55^ to determine the methodological quality, rigor, trustworthiness, relevance, and results of all included studies according to their research method (ie, quasiexperimental, qualitative, case‐controlled, cross‐sectional, and review studies). Two authors independently assessed the methodological quality of studies, and any discrepancies between reviewers were resolved collaboratively through discussion.

### Data Extraction

A Microsoft Excel data extraction form was developed and piloted for extracting data from eligible studies to identify sections relevant to the research question and to analyze the data. The following variables were extracted: authors, title, year published, ethics, study aim and objectives, sample size, participants characteristics, study design, study location, setting, data collection period, data collection, analysis, and variables of interest such as type of trauma and how pregnant women experienced trauma or retraumatization within maternity services. 

### Data Analysis and Synthesis

The narrative approach to synthesis uses words and text from the findings to tell the story. Popay et al's^56^ 4‐stage framework was followed as it increases the transparency and trustworthiness of the narrative synthesis and involves describing, organizing, exploring, and interpreting the study findings, taking into account the methodological adequacy. This was done by investigating both similarities and differences between findings, including study design, quality, study power, participant characteristics, experiences of trauma and retraumatization, and outcome measures. This enabled the identification of analytic themes relevant to the review question.

## RESULTS

### Search Outcomes

**Figure 1:**
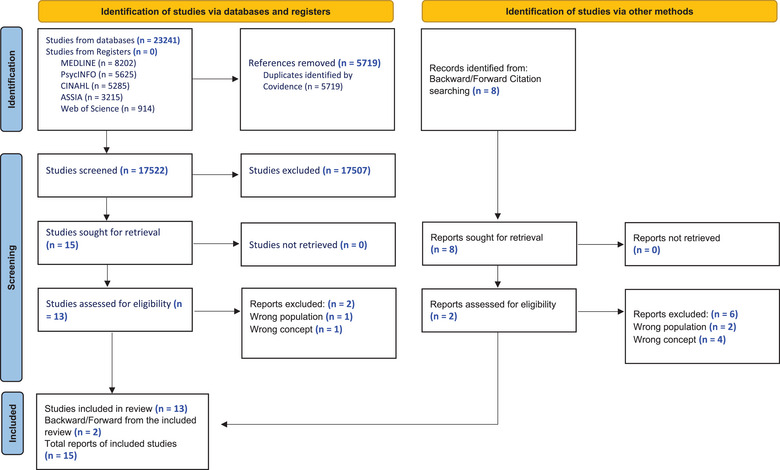
Preferred Reporting Items for Systematic Reviews and Meta‐Analyses Statement 2020 Flowchart of the Study Selection Process

The search strategy yielded 23,241 studies from 5 databases; duplicates were removed (n = 5719), and 17,522 studies were retained for title and abstract screening. A total of 15 full‐text papers were screened, and 13 papers met the inclusion criteria (see Figure 1). Handsearching backward and forward citations yielded 8 additional studies. Following full‐text review, 2 of these studies were deemed eligible, yielding a final total of 15 studies for this review. Table 1 presents summary information of the included studies and main findings.

**Table 1:** Summary of Included Studies (Study Characteristics and Key Results)

Author and Country	Study Methods	Study Population	Data Analysis	Key Results
Leeners et al^63^ Germany	Quantitative study. Data collected via modified questionnaire (Wyatt, 1998) along with semistructured interviews.	Participants: n = 85 anonymous women who were compared with n = 170 women with no CSA history (control group).	*t* test and Mann‐Whitney tests as well as χ^2^ tests, Fisher's exact test, and multiple logistic regression analysis.	Activators for participants: (1) Vaginal and cervical examinations, (2) male HCPs, (3) loss of control, (4) nakedness and touch/being looked at, (5) duration and unpredictability of labor, (6) position in labor, being asked to lay down in a particular position. Trauma outcomes: The following consequences of memories of CSA on the labor process were described by women: feelings of not being able to relax (54.3%), increase in pain (31.4%), difficulty in cooperating with the midwife (31.4%), increased duration of labor (28.6%), panic (28.6%), difficulty in relaxing (25.7%), cessation of contractions (20.0%), and impossibility of vaginal examinations (5.7%). Additionally, panic, fear, shortness of breath, and sickness were experienced by some women following memories of CSA. Participants experiences: Memories of the original abuse situation during the birth arose and disturbed birth in 41.2% of the women with CSA. In 9.7% of these women, memories reappeared for the first time since childhood. Of women (n = 49) who experienced dissociation, 36.7% considered this experience exclusively as helpful and based this on using dissociation to reduce pain (59.1%) as well as fear (4.5%) and to reduce overwhelming aspects of the birth and to gain control of the situation (40.9%).Women who experienced (10.2%) exclusively as disturbing based this on a sudden loss of contact with perinatal health care providers. What participants found helpful or reduced/prevented distress or (re)traumatization: Participation in decision‐making was related to an increase in satisfaction. Fear reduction was predicted by feeling adequately prepared for labor as well as participation in medical decision‐making. HCPs being trained to address and talk about complications and those who are survivors of CSA.
Lev‐Wiesel et al^67^ Israel	Quantitative study via self‐report questionnaire.	Participants: n = 323 (CSA), n = 868 (1 traumatic event), n = 152 (no history of assault).	Convenience sampling. Univariate analysis of variance, analysis of covariance.	Activators for participants: Intrusion and arousal following childbirth led to an increase in retraumatization for CSA survivors. Trauma outcomes: CSA survivors scored higher at all data collection time points. Women in the CSA group scored higher on their total PTSD score, avoidance level, and in the dissociation score than no‐trauma group and other‐trauma group. Women in the CSA group had higher level of arousal (F_2,832_ = 5.64; *P* < .01) and higher level of total PTSD (F_2,664_ = 26.30; *P* < .001) compared with both comparison groups. Women in the CSA group had increased PTSD subcategories of intrusion and arousal following childbirth, although the overall PTSD score did not increase following childbirth in any of the groups. Participants experiences: Not stated. What participants found helpful or reduced/prevented distress or (re)traumatization: Not stated.
Leeners et al^62^ Germany	Quantitative study. Data collected via Microsoft Access Database. Face‐to‐face semistructured interviews.	Participants: n = 85 anonymous women, n = 170 women with no history of CSA.	*t* tests and Mann‐Whitney tests were carried out to compare groups. Multiple logistic regression analysis..	Activators for participants: (1) Vaginal examinations, (2) general medical examinations, (2) realization of impending of motherhood, (3) tiredness and morning sickness, (4) having to get support from others, (5) physical changes, (6) communicating with HCPs, (7) second and third trimester were seen to be the most activating for women who suffered CSA, (8) prenatal classes. Trauma outcomes: CSA experiences activated memories during pregnancy. Participants experiences: 20% of women (n = 17) felt increased fear regarding pregnancy and birth. Some women had difficulties in sensing their own body throughout pregnancy, and others could not adequately acknowledge and realize their own needs and mentioned an impaired confidence toward medical professionals. Dissatisfaction was mainly attributed to feeling ignored (17.4%, n = 4), disrespected (17.4 %, n = 4), and not being offered the opportunity to discuss CSA (26.1 %, n = 6). What participants found helpful or reduced/prevented distress or (re)traumatization: Attentive and respectful HCPs, competence in HCPs for the history of abuse survivors and the consequences of that. Emotional support such as being empathetic, understanding, sensible, confident, and not being pressed for time. 12.2% of women (n = 5) suggested improved training of physicians and other health care providers regarding the consequences of CSA specifically in perinatal situations.
Rhodes et al^57^ United States	Qualitative tudy. Data collected s private interviews.	Women (n = 7) sexual abuse survivors; nurses or midwives (n = 5); labor and delivery nurses (n = 3).	Data analysis: domain analysis, taxonomic analysis, componential analysis, and validity testing.	Activators for participants: (1) Labor itself, (2) positions in labor, (3) position they were placed in for birth, (4) difficulty pushing during labor, (5) mistrust in their own body. Trauma outcomes: Activating thoughts led to sensory information—bodily sensations. Participants experiences: “I was spread‐eagled on a bed … it brought back the bondage.” What participants found helpful or reduced/prevented distress or (re)traumatization: Keeping the participants focused and grounded stops the activating thoughts and memories—the HCP's voice can help visualize and understand what is happening
Sobel et al^48^ United States	Qualitative study using interviews.	Women (n = 20) with a history of sexual trauma who had given birth in past 3 y and have experienced sexual violence, abuse, or rape; and women (n = 10) with no trauma history	Thematic analysis using NVivo 11. Grounded theory, an inductive methodology.	Activators for participants: (1) Language and communication from HCPs, (2) having no partner or loved one in the room. Trauma outcomes: Not stated. Participants experiences: HCP‐“I'm gonna check you … better not move.” What participants found helpful or reduced/prevented distress or (re)traumatization: Positive language “You can do it,” no negative words or outwardly addressing rape history or using the word *rape*
Coles and Jones^68^ Australia	Qualitative study. Semistructured, in‐depth interviews.	Women (n = 11) who self‐identified as sexually abused by a family member were interviewed in the first group. Women (n = 7) in the second group to further explore the primary care needs of postpartum women after the initial study.	Thematic analysis using NVivo 2.0 and 7.0 software.	Activators for participants: (1) Intimate examinations, (2) physical examinations of their newborn, (3) male HCPs, (4) lying on the flat of their back with no undergarments on (positions in labor). Trauma outcomes: Feelings of anger toward themselves and others, blame, shame and guilt. Feelings of concerns about handing over their newborn to a man. Participants experiences: Not stated. What participants found helpful or reduced/prevented distress or (re)traumatization: Participants suggested that HCPs should create a rapport with patient, HCPs should have knowledge of trauma and trauma‐informed care and perinatal services.
Halvorsen et al^64^ Norway	Qualitative study. Semistructured interviews.	Women (n = 10) who had been exposed to rape after the legal age of consent (≥16 y) and before giving birth to their first child.	Thematic analysis.	Activators for participants: (1) Legs being splayed apart, (2) invasive procedures, (3) being undressed, (4) unfamiliar hands touching their body, (5) HCPs taking control. Trauma outcomes: Feelings of escapism, objectification and feelings of being *dirty* and feelings of alienation. Participants experiences: Described their childbirth as a “new assault.” What participants found helpful or reduced/prevented distress or (re)traumatization: Creating a safe and calm space for the participant to have birth. Having the birth attendant being aware of the history of the trauma.
Nerum et al^65^ Norway	Quantitative study. Interviews using conversational approach.	Primiparous women (n = 373): 185 subjected to CSA, 47 subjected to RA, 141 in control group with no history of abuse.	Case‐control study in clinical cohort. The χ^2^ tests and the Kruskal‐Wallis tests were applied. Data were analyzed through SPSS 19.0 and STAT 12.	Activators for participants: Not stated. Trauma outcomes: The RA group showed a significantly higher risk for caesarean (adjusted OR, 9.9; 95% CI, 3.4‐29.4) and operative vaginal birth (adjusted OR, 12.2; 95% CI, 4.4‐33.7) compared with the control group. There were no significant differences between women in the CSA and the control group. The RA group displayed significantly longer duration in all stages of labor as compared with the control and CSA groups. Participants experiences: Not stated. What participants found helpful or reduced/prevented distress or (re)traumatization: Not stated.
Byrne et al^61^ United Kingdom	Qualitative narrative research. Participants were invited to 3 open‐ended interviews.	3 women having experienced CSA from single or multiple perpetrators, family or nonfamily, before the age of 16 participated in 3 open‐ended interviews.	Narrative summaries were used to identify the narrative themes.	Activators for participants: (1) Breastfeeding, (2) being “body focused,” (3) no control, (4) vaginal examinations, (5) sexual activities with partner after birth, (6) giving birth to boy. Trauma outcomes: All participants were attending mental health services. Participants experiences: Participants felt “trapped between ‘mental health’ and ‘normal.’” What participants found helpful or reduced/prevented distress or (re)traumatization: Normalizing breasts made the participants feel more comfortable, and when HCPs made examinations positive instead of negative, it made the idea of examinations less daunting.
Records and Rice^58^ United States	Qualitative study. Semistructured private interviews.	7 Hispanic women with a history of abuse Hispanic women was recruited from a rural prenatal clinic.	Psychophenomonological method was used to analyze the women's experiences.	Activators for participants: (1) No support, (2) not being able to make sense of the pain. Trauma outcomes: Feelings of fear, being scared, worried, depressed, panicked, and anxious. Participants experiences: Not stated. What participants found helpful or reduced/prevented distress or (re)traumatization: Not stated.
LoGiudice and Beck^30^ United States	A qualitative descriptive phenomenological research design. Private interviews.	8 women self‐identified as survivors of sexual abuse with at least one childbearing experience.	A descriptive phenomenology was used to analyze the women's experiences.	Activators for participants: (1) Not asking women about their history, (2) HCPs not engaging with their patient and not noticing obvious signs of something not being right, (3) intimate procedures such as fingers near genitalia for examinations and cervical checks/dilation analysis, (4) HCPs placing newborn on their chest, (5) child being placed for adoption and staff not addressing it. Trauma outcomes: Not being supported by HCPs—felt symptoms of PTSD, depression, anxiety, fear, nervousness and unpleasantness. Participants experiences: “this slimy, squirmy thing”—child being placed on them. What participants found helpful or reduced/prevented distress or (re)traumatization: Screening for history, therapeutic relationship, delivery of care, HCPs not addressing how many fingers the patient has dilated.
Stevens et al^59^ United States	Quantitative study.	Participants were pregnant patients (n = 41) receiving care at an outpatient perinatal clinic within a large urban teaching hospital in Chicago, Illinois. Participants were <30 wk pregnant and experienced at least one incident of physical, sexual, or emotional abuse, , or “violent” physical or sexual assault.	Frequency analysis, Clinical cutoff scores for CTQ, PCL‐C, and PHQ‐9, and correlations were computed.	Activators for participants: Not stated. Trauma outcomes: Majority of participants (82.9%) reported at least one past history of at least one incident of physical, sexual, or emotional abuse at the eligibility screening. At least one type of childhood abuse was reported by participants (65.9%). Obstetricians detected abuse histories in less than one‐quarter of cases, almost half of participants (46%) received invasive examinations for nonroutine reasons. Perinatal care can be an especially stressful experience for women who have a history of abuse. PTS and depression symptoms were associated with lower self‐efficacy in communicating perinatal care preferences. Participants experiences: Not stated. What participants found helpful or reduced/prevented distress or (re)traumatization: Screening for past abuse.
Jonsdottir et al^66^ Iceland	Qualitative phenomenological study. 16 in‐depth interviews were conducted.	9 women CSA survivors. 7 of the women reported first occurrence of sexual violence at preschool or elementary school age, and 2 reported sexual violence in adolescence. 2 women were victims of relatives, and most of the women were repeatedly abused.	Thematic analysis and analytical framework was constructed where the main themes and subthemes were presented.	Activators for participants: (1) Lack of control, (2) overall abuse of power from HCPs, (3) vaginal examinations, (4) no explanation from HCPs as to what was going on. Trauma outcomes: Feeling of shame, guilt, embarrassment and unbearable. Participants experiences: Not stated. What participants found helpful or reduced/prevented distress or (re)traumatization: Screening for past experiences of abuse.
Kitzinger^45^ United Kingdom	Qualitative study.	Women (n = 39) who identified as survivors of CSA (mainly incest).	Data analysis: not reported.	Activators for participants: (1) Physical examinations, (2) labor and birth were perceived as traumatizing, (3) act of pushing through contractions while laying on their back. Trauma outcomes: Feeling skewered and abused. Participants experiences: Feeling like “a lump of meat.” What participants found helpful or reduced/prevented distress or (re)traumatization: Holding the newborn helped them distract from their abuse history, as well as knowledge about their genitalia, and being able to ask midwife questions helped them.
Montgomery^60^ United Kingdom	Qualitative synthesis	The 8 included qualitative studies reported results from a total of 126 women who all had experienced CSA.	Data analysis: meta‐synthesis of 8 qualitative studies.	Activators for participants: (1) Physical examinations particularly relating to touch or intimate examination, which also includes examination of the newborn, (2) sensory activators such as smells and sounds, (3) lack of control activated distressing memories or flashbacks, (4) fear accompanies flashbacks, especially if women cannot contextualize them, (5) male HCPs. Trauma outcomes: Dissociation, avoidance of HCPs, vulnerability and feelings of *fright‐flight‐fight* mode. Participants experiences: Not stated. What participants found helpful or reduced/prevented distress or (re)traumatization: Privacy and control helps the patient feel safe.

Abbreviations: CSA, child sexual abuse; CTQ, Childhood Trauma Questionnaire; HCP, health care professional; PCL‐C, PTSD CheckList‐Civilian Version; PHQ‐9, Patient Health Questionnaire‐9; PTSD, posttraumatic stress disorder; RA, rape in adulthood.

### Study Characteristics

Of the 15 studies, 5 were in the United States,^30^, ^48^, ^57^, ^58^, ^59^ 3 were conducted in the United Kingdom,^45^, ^60^, ^61^ 2 were in Germany,^62^, ^63^ 2 were in Norway,^64^, ^65^ and 1 each was in Iceland,^66^ Israel,^67^ and Australia.^68^ Five studies used a quantitative approach,^57^, ^59^, ^63^, ^65^, ^67^ 9 studies used a qualitative approach,^30^, ^45^, ^48^, ^57^, ^58^, ^61^, ^64^, ^66^, ^68^ and 1 study used qualitative synthesis.^60^ Three of the quantitative studies used a cohort study design,^62^, ^63^, ^67^ one used a case‐control study design,^65^ and one used a quasiexperimental study design.^59^ Individual participant interviews were used in all of the qualitative studies.^30^, ^45^, ^48^, ^57^, ^58^, ^61^, ^64^, ^66^, ^68^ The included studies reported results from a total of 1884 participants. All participants were identified as female and aged 18 or older, with an age range of 18 to 58. Fourteen studies included only English‐speaking participants, and one study^58^ included both Spanish and English speaking participants. All participants had previously given birth and reported a history of CSA, rape, or sexual abuse.

### Quality Appraisal

The JBI Checklist^55^ was used to assess the overall quality appraisal of the 15 included studies (see Supporting Information: Appendix S2). All the studies met the checklist criteria, leading to all studies being included in the review. The 3 cohort studies^62^, ^63^, ^67^ were unclear on their strategies to address incomplete follow‐up.

### Thematic Analysis

Data Analysis produced 3 themes with supporting subthemes. Theme 1, activating events, had 4 subthemes: positions in labor, intimate procedures, communications with HCPs, and loss of control. Theme 2, outcomes, had one subtheme: emotional responses. Theme 3, interventions reducing or preventing retraumatization, had 2 subthemes: role of the HCP and screening for abuse and history of trauma.

**Figure 2:**
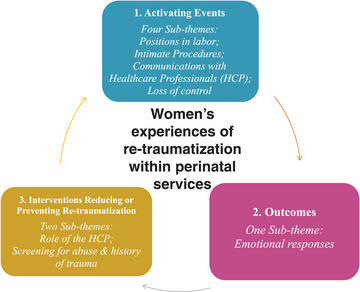
Representation of Women's Experiences of Retraumatization Within Perinatal Services

To illustrate synthesis, the main themes and their supporting subthemes are presented with narratives explicating the contribution of the subthemes to the overall understanding of participants’ experiences. In addition, a thematic summary diagram is provided in Figure 2, a summary of supporting quotations from across the studies is provided in Table 1, and detailed information on the analytic themes and supporting quotations is provided in Supporting Information: Appendix S3.

### Activating Events

Activating events were the combined impact of being placed in retraumatizing positions, being subject to intimate procedures and examinations, the compounding effect of insensitive or inadequate professional communication, and not feeling in control contributing to the experience of retraumatization.

#### Positions in Labor

Five studies^57^, ^62^, ^63^, ^64^, ^68^ clearly reported that the positions women were placed in for labor was retraumatizing, with one participant quoted as saying, “I was spread‐eagled on a bed … it brought back the bondage.”^57^ Included studies indicated that lack of consultation and lack of choice in positioning were particularly activating.^63^, ^67^

#### Intimate Procedures

Nine papers found that intimate procedures such as vaginal examinations and cervical dilation assessments to be activating.^30^, ^60^, ^61^, ^62^, ^63^, ^64^, ^66^, ^68^ Lack of control, perceived abuse of power, and lack of support around intimate procedures were particularly noted in included studies.^61^, ^68^ Jonsdottir et al^66^ concluded that although all women may have been subject to CSA, not all are willing to disclose this. Therefore, service providers must routinely ask permission and include women in decision‐making.

#### Communications With HCPs

Three studies^48^, ^64^, ^66^ found that communication with HCPs, or the lack of communication, was a cause for activating thoughts or memories for the participant. Sobel et al^48^ interviewed 20 women who self‐identified as having a history of sexual trauma. These participants stated that the HCPs used activating language such as, “I'm gonna check you … better not move.”^48^

Other qualitative studies found the communications with HCPs and the language used was traumatizing.^30^, ^48^, ^61^ Another common activate was the lack of control participants felt when it came to their labor and birth.^61^, ^66^

#### Loss of Control

Two studies specified that loss of control for the participants was activating and retraumatized them.^64^, ^66^ Participants specifically noted HCPs failed to justify their actions and decisions, leading to feelings of guilt and shame. Three qualitative studies^45^, ^57^, ^68^ found that the position in which women were placed in for their examinations and labor without discussion were activating. In particular, participants experienced their vaginal and cervical examinations to be retraumatizing and cause flashbacks.

### Outcomes

Outcomes illustrated how participants in the included studies identified that the outcomes from retraumatizing were emotional, namely feelings of anger, blame, panic, anxiety, or guilt.^30^, ^58^, ^66^, ^68^ These outcomes reflected the nascent stage of trauma awareness in clinical settings and the lack of formal trauma screening.

#### Emotional Responses

Emotional responses such as dissociation, escapism, shame, blame, guilt, or feelings of being *dirty* were reported in 9 of the reviewed studies.^30^, ^45^, ^58^, ^60^, ^62^, ^63^, ^64^, ^66^, ^68^ Kitzinger stated that one participated felt “skewered and abused,” which led them to feel like “a lump of meat.”^45^ Similarly, Halvorsen et al^64^ identified that participants were retraumatized by the HCPs’ failure to develop rapport before physical interventions, leading to feelings of shame and guilt.

### Interventions Reducing or Preventing Retraumatization

Participants in several studies described strategies that reduced or prevented retraumatization and saw early communication as a protection against retraumatization. Jonsdottir et al^66^ suggested HCPs build rapport and offer advice in a supportive manner. LoGiudice and Beck's^30^ qualitative study identified that when HCPs were able to give women with a history of sexual abuse choices (eg, infant feeding), this created a more comfortable relationship with the HCP.

#### Role of the HCP

The role of the HCP is crucial in reducing or increasing the likelihood of (re)traumatization for women in maternity services. Study participants outlined the importance of aspects of positive language such as “you can do it” and not using the term *rape*, which can greatly reduce the (re)traumatization.^30^, ^45^, ^48^, ^61^, ^62^, ^63^, ^66^, ^68^ Likewise, Sobel et al^48^ stated that participants felt consistency of contact was important while establishing a trusting relationship. They also noted that flexible HCPs who could quickly react to crisis and individual needs were valuable in reducing retraumatization. Leeners et al^63^ concluded that when HCAs created a trusting environment, women's experiences of CSA could be acknowledged and ameliorated.

#### Screening for Abuse and History of Trauma

Seven studies^30^, ^59^, ^62^, ^63^, ^64^, ^66^, ^68^ highlighted the importance of screening for past abuse. Screening for past abuse was seen as helpful, as it can help identify trauma and create a safe positive environment for women as they experience childbirth, with a view to preventing retraumatization. Many participants reported that if the HCP had prior knowledge of their abuse, it would have helped reduce their trauma.^64^, ^68^ Establishing routine screening may lead to a significant reduction of (re)traumatization.^59^, ^67^ However, other studies^59^, ^62^, ^63^, ^67^ reported a lack of routine screening by HCPs for a prior history of abuse or trauma. These studies concluded that HCPs need to be willing to ask about past abuse and knowledgeable in how to deal with the consequences of such experiences, as this can be the first step to preventing any potential unintended retraumatization. These studies also indicated that this gap is not due to a lack of reliable tools but instead requires a cultural shift whereby perinatal services routinely prioritize assessment of women exposed to past abuse and trauma.

## DISCUSSION

This is the first integrative review to analyze the experience of retraumatization in perinatal services. These findings offer insight into the experiences of women who have a history of trauma, and may therefore be retraumatized through perinatal care, and those who may be traumatized through perinatal care delivery regardless of past history. This evidence supports the premise that women in the perinatal period are likely to have a history of previous trauma that may be underreported and undetected and thus experience retraumatization in perinatal care settings. Similar to results from other studies, physical (pelvic) examinations, positioning in labor, and feeling a loss of control were the most commonly reported activators of retraumatization.^30^, ^31^, ^39^, ^42^ These findings highlight the urgent need for TIC education in perinatal care settings for all staff and providers. Adopting universal screening protocols at the first prenatal visit for any past history of ACE, sexual assault, or abuse will then facilitate the development of TIC principles and practices within perinatal care models.^19^, ^28^, ^32^, ^38^, ^39^, ^69^

This review identifies TIC as one approach that can improve care for those who have experienced trauma ranging from ACEs to sexual assault.^19^, ^27^, ^28^, ^32^, ^38^ The framework for TIC was created by SAMHSA^27^ to improve care provided to survivors of physical and sexual violence and substance use disorders, aiming to promote safety and prevent any potential unintended retraumatization. TIC should not be viewed as additional work; instead it should be implemented into everyday practice by helping HCPs apply the core principles of TIC.^28^ Subsequently application of TIC in the perinatal period addresses the importance of advocating for patients, and recognizing that any experience, whether it be in the labor room, or checking in for an appointment, has the ability to activate and impact care.^28^, ^38^, ^46^, ^47^, ^48^ TIC mitigates retraumatization by promoting women's autonomy. Thus, TIC is empowering as it promotes women's capacity and participation in shared decision‐making by asking for permission to proceed or to stop.

### Strengths and Limitations

This study has notable strengths. This is the first integrative review that addressed the issue of retraumatization in the perinatal period by drawing on the experiences of 1884 women from a range of geographic locations who reported a prior history of trauma (sexual abuse, rape). Rigor was improved by the use of Russell's^50^ 5‐stage integrative review process and Popay et al's^56^ 4‐stage framework, which increased the transparency and trustworthiness of the narrative synthesis. Our results strengthen the evidence base for this important topic and fills a gap in the literature. Although participants from the included studies were from various backgrounds and geographic locations, many similarities were seen in their experiences of retraumatization in perinatal services. Participants highlighted their concerns in relation to the lack of TIC and screening of past history (of abuse) and reiterated that this is the way forward for perinatal services. Certain limitations are also evident in this review. Given the inclusion criteria and methodological limitations, it is possible that relevant studies were not identified and may have been omitted. There was also a noted lack of recent randomized controlled trials conducted on this topic, which challenges the true impact on the findings.

### Implications for Health Care Policy and Practice

There are a number of strategies that can be used to effectively implement TIC in perinatal services to reduce the risk of retraumatization of women in the perinatal period. These include educating all staff in TIC and having leadership within perinatal services that is aware of and committed to TIC in the screening, including screening for trauma, planning, implementing, and evaluating TIC delivery. There is also a need to consult with midwifery staff and women in the perinatal period, contributing to the understanding of the impact of procedures such as positions in labor, intimate examinations, communications with HCPs, and experience of loss of control. Acknowledging these common activators relating to women's past history of trauma and retraumatization can inform future compassionate practices in reducing these experiences in perinatal services. Universal screening for trauma histories at the initial point of care would assist midwives and perinatal staff in planning for collaborative TIC. This can be done by encouraging women in the perinatal period to complete patient satisfaction surveys and feedback forms after discharge as this would inform policy making and local procedures on TIC. Improved education and training for midwives and all other perinatal staff could include routine screening questions about trauma or abuse when taking a woman's history. This would facilitate more empathetic support and increase HCPs’ confidence in adapting to the specific needs of women with history of trauma. Central to normalizing TIC is educating HCPs on effective screening for and responses to past trauma. Such training for HCPs and staff is needed for use as part of initial patient registration and could be included in ongoing professional development. Midwives and perinatal staff need to be confident in their approach and competent in their knowledge, as well as understand the risk of retraumatization for women during the perinatal period. Physical environments and procedures during perinatal services need to be welcoming, compassionate, and empowering and collaboratively involve women in individualized TIC plans.

### Implications for Future Research

More research is needed to identify how health care providers are specifically screening and assessing for a history of trauma during perinatal care. Such evidence could inform how HCPs understand the language used to respond to individual's concerns, how to develop more thoughtful therapeutic relationships, and the nature of those invasive procedures and intimate examinations that are particularly challenging. Choice and safety are promoted to prevent any potential unintended retraumatization. Future studies are needed to develop TIC curricula for undergraduate nursing, midwifery, and medical education. This includes postgraduate studies that assess TIC educational competencies through simulation training, which would allow learners to practice conversations and interventions related to trauma. Practice standards can then serve as a foundation for providing high‐quality, patient‐centered care and better outcomes to the increasing number of people with history of trauma.

## CONCLUSION

This review provides evidence that clearly links trauma, abuse, and birth‐related trauma to women's experiences of retraumatization in perinatal services and offers insight into improvements to help women to feel safe and protected in perinatal services. This review has highlighted an urgent need for all hospitals to respond and acknowledge women in the perinatal period who have histories of trauma and require trauma‐informed perinatal care. Women in the perinatal period may have histories of trauma and vulnerabilities that are often overlooked or underreported. HCPs in perinatal services should work from the premise that all women may be subject to trauma and should thus provide a safe space to develop individualized compassionate TIC care plans. All women, including those with a history of trauma, need TIC‐informed communication and inclusion plus similarly informed emotional and physical support with access to mental health interventions.

## CONFLICT OF INTEREST

The authors have no conflicts of interest to disclose.

## Supporting information


**Appendix S1**. Search Strategy

**Appendix S2**. Quality Appraisal

**Appendix S3**. Additional Information on Analytic Themes and Supporting Quotations
