# Supplementary material for: An Integrative Review Exploring Womens’ Experiences of Retraumatization Within Perinatal Services
Source: J Midwifery Womens Health. 2024 Jul 22;70(1):32–49. doi: 10.1111/jmwh.13662 (PMC11803493; doi:10.1111/jmwh.13662)
Supplement: Supplementary file 1 — Appendix S1. Search Strategy [file JMWH-70-32-s002.docx]

***Supplementary Appendix 1: Search Strategy***

| ***Population***  Women during pregnancy | women OR pregnan* OR matern* OR mother* OR childbirth OR childbearing  AND |
| --- | --- |
| ***Concept***  Experiences of re-traumatisation | MM "Child Abuse" OR DE "Child Neglect" OR MM "Trauma-Informed Care" OR MM "Traumatic Experiences" OR DE "Stress" OR MM "Traumatic Experiences" OR MM "Experiences (Events)" OR MM "Trauma" OR MM "Collective Trauma" OR MM "Posttraumatic Stress" OR MM "Posttraumatic Stress Disorder" OR MM "Trauma Reactions" OR MM "Trauma Screening" OR MM "Trauma Treatment" OR retraumatise OR retraumatize OR retraumatisation OR retraumatization OR re-traumatisation OR re-traumatization OR Neglect* OR Sexual abuse OR sexually abus* OR sexual abus* OR abuse* OR advers* OR Adverse childhood experience OR ACE OR Victim* violence OR maltreat* OR child maltreatment OR mistreat OR emotional abuse OR psychological abuse OR PTSD OR post traumatic stress OR post-traumatic stress OR post- traumatic* OR PTSS OR posttrauma* OR trauma* OR traumatic life event* OR psychological trauma OR stress disorder* OR stress reactions OR Complex PTSD OR Complex Trauma  AND |
| ***Context***  Maternity care services | maternal health services OR maternity service* OR maternity care OR obstetric service OR perinatal OR peri natal OR peri-natal OR antenatal OR postnatal OR postpartum OR prenatal OR postpartum OR birth* OR labour |
