# Supplementary material for: An Integrative Review Exploring Womens’ Experiences of Retraumatization Within Perinatal Services
Source: J Midwifery Womens Health. 2024 Jul 22;70(1):32–49. doi: 10.1111/jmwh.13662 (PMC11803493; doi:10.1111/jmwh.13662)
Supplement: Supplementary file 2 — Appendix S2. Quality Appraisal [file JMWH-70-32-s003.docx]

***Supplementary Appendix 2: Quality Appraisal***

| **Joanna Briggs Institute: Qualitative Research Checklist** | **Rhodes et al. 1994** | **Sobel et al. 2018** | **Coles et al. 2009** | **Halvorsen et al. 2013** | **Byrne et al. 2017** | **Records & Rice, 2002** | **LoGiudice & Beck, 2016** | **Jonsdottir et al. 2020** |
| --- | --- | --- | --- | --- | --- | --- | --- | --- |
| Is there congruity between the stated philosophical perspective and the research methodology? | **YES** | **YES** | **YES** | **YES** | **YES** | **YES** | **YES** | **YES** |
| Is there congruity between the research methodology and the research question or objectives? | **YES** | **YES** | **YES** | **YES** | **YES** | **YES** | **YES** | **YES** |
| Is there congruity between the research methodology and the methods used to collect data? | **YES** | **YES** | **YES** | **YES** | **YES** | **YES** | **YES** | **YES** |
| Is there congruity between the research methodology and the representation and analysis of data? | **YES** | **YES** | **YES** | **YES** | **YES** | **YES** | **YES** | **YES** |
| Is there congruity between the research methodology and the interpretation of results? | **YES** | **YES** | **YES** | **YES** | **YES** | **YES** | **YES** | **YES** |
| Is there a statement locating the researcher culturally or theoretically? | **YES** | **YES** | **YES** | **YES** | **YES** | **YES** | **YES** | **YES** |
| Is the influence of the researcher on the research, and vice-versa, addressed? | **YES** | **YES** | **YES** | **YES** | **YES** | **YES** | **YES** | **YES** |
| Are the participants, and their voices adequately represented? | **YES** | **YES** | **YES** | **YES** | **YES** | **YES** | **YES** | **YES** |
| Is the research ethical according to current criteria or, for recent studies, and is there evidence of ethical approval by an appropriate body? | **YES** | **YES** | **YES** | **YES** | **YES** | **YES** | **YES** | **YES** |
| Do the conclusions drawn in the research report flow from the analysis, or interpretation, of the data? | **YES** | **YES** | **YES** | **YES** | **YES** | **YES** | **YES** | **YES** |

| **Joanna Briggs Institute: Quasi-Experimental Studies Checklist** | **Stevens et al. 2016** |  |  |
| --- | --- | --- | --- |
| Is it clear what is the ‘cause’ and what is the ‘effect’? | **YES** |  |  |
| Were the participants included in any comparisons similar? | **N/A** |  |  |
| Were participants included in any comparisons receiving similar treatment/care, other than the exposure or intervention of interest? | **NO** |  |  |
| Was there a control group? | **NO** |  |  |
| Were there multiple pre and post measurements of the outcome? | **NO** |  |  |
| Was follow up complete and if not, were differences between groups in terms of their follow up adequately described and analyzed? | **NO** |  |  |
| Were the outcomes included in any comparisons measured in the same way? | **NO** |  |  |
| Were outcomes measured in a reliable way? | **YES** |  |  |
| Was appropriate statistical analysis used? | **YES** |  |  |

| **Joanna Briggs Institute: Case Control Studies Checklist** | **Nerum et al. 2012** |  |  |
| --- | --- | --- | --- |
| Were the groups comparable other than the presence of disease in cases or the absence of disease in controls? | **YES** |  |  |
| Were cases and controls matched appropriately? | **YES** |  |  |
| Were the same criteria used for identification of cases and controls? | **YES** |  |  |
| Was exposure measured in a standard, valid and reliable way? | **YES** |  |  |
| Was exposure measured in the same way for cases and controls? | **YES** |  |  |
| Were confounding factors identified? | **YES** |  |  |
| Were strategies to deal with confounding factors stated? | **Unclear** |  |  |
| Were outcomes assessed in a standard, valid and reliable way for cases and controls? | **YES** |  |  |
| Was the exposure period of interest long enough to be meaningful? | **YES** |  |  |
| Was appropriate statistical analysis used? | **YES** |  |  |

| **Joanna Briggs Institute: Cohort Studies Checklist** | **Leeners et al. 2016** | **Leeners et al. 2013** | **Lev-Wiesel et al. 2009** |
| --- | --- | --- | --- |
| Were the two groups similar and recruited from the same population? | **YES** | **YES** | **YES** |
| Were the exposures measured similarly to assign people to both exposed and unexposed groups? | **YES** | **YES** | **YES** |
| Was the exposure measured in a valid and reliable way? | **YES** | **YES** | **NO** |
| Were confounding factors identified? | **YES** | **YES** | **NO** |
| Were strategies to deal with confounding factors stated? | **YES** | **YES** | **NO** |
| Were the groups/participants free of the outcome at the start of the study (or at the moment of exposure)? | **YES** | **YES** | **YES** |
| Were the outcomes measured in a valid and reliable way? | **YES** | **YES** | **YES** |
| Was the follow up time reported and sufficient to be long enough for outcomes to occur? | **YES** | **YES** | **YES** |
| Was follow up complete, and if not, were the reasons to loss to follow up described and explored? | **YES** | **YES** | **YES** |
