# Supplementary material for: An Integrative Review Exploring Womens’ Experiences of Retraumatization Within Perinatal Services
Source: J Midwifery Womens Health. 2024 Jul 22;70(1):32–49. doi: 10.1111/jmwh.13662 (PMC11803493; doi:10.1111/jmwh.13662)
Supplement: Supplementary file 3 — Appendix S3. Additional Information on Analytic Themes and Supporting Quotations [file JMWH-70-32-s001.docx]

| ***Appendix 3: Additional Information on Analytic Themes and Supporting Quotations:*** | |
| --- | --- |
| **Theme and Sub Themes** | **Supporting Quotations** |
| **Participants experience of interventions helpful in reducing or preventing (re)-traumatization (sub-themes**  **Two Sub-themes: Role of the Healthcare Professional, Screening for abuse and history of trauma.** | “She was great! She was also a psychiatric nurse; so, she went through a lot of this experience with me, and she kind of talked me out of this stiffness towards pregnancy and birth” (Jonsdottir *et al*. 2020 p.678)  “She was just wonderful. She was very vigilant about it and gave me great advice. It just worked really well.” (Jonsdottir *et al*. 2020 p.680)  “I do remember there was one nurse that was, I guess she was doing the birth certificate or something, she did ask me who the father was. And I can still remember clear as a bell, I said I didn’t know, even though I knew perfectly well. I just couldn’t deal with it, so I said I don’t know who it is. I distinctly remember hearing her muddle under her breath, ‘oh, irresponsible kid’. I was just (thinking) … Oh my god, I am irresponsible. Who does this?” (Jonsdottir *et al.* 2020 p.478)  “Developing a really good relationship, like when you trust the GP and where you see the same GP, that’s really good, being able to go overtime with a GP if you’re in crisis or a bit distressed … not having them constantly sort of check their clock and things. A GP who will sneak you into the queue because they’ve noticed that you’re crying, they’re concerned that you’re suicidal, so they let you jump ahead of ten other people. Things like that, just going beyond the call… just little things like that, they are the things that you notice” (Penny) (Sobel *et al*. 2018)  “That the consistency of care and relationships developed were really crucial” (Sarah) (Sobel *et al.* 2018)  “She comes to me … really nice and says, ‘I was reading… about your experience (of CSA)’ because I had stated that I had experienced sexual-violence, and (she continues), ‘let’s just go very slowly and carefully, and if you allow me, I would like to examine you, the dilation, and how you are doing, and then if you are willing to, then I will start the drip again’. This was great experience because I had a say in the matter” (Jonsdottir *et al*.  2020 p.679)  “My doctor was a female OB and she knew I was going to give up the baby for adoption but she never, she never asked any questions. I mean I don’t know whether she thought it was like none of her business or whatever, but she never asked who the father was, never asked if I was a sexual abuse survivor, none of that.” (LoGiudice and Beck 2016 p.477)  “She was just wonderful. She was very vigilant about it and gave me great advice. It just worked really well.” (Jonsdottir *et al*. 2020 p.680)  “There was a nurse there, but she never asked me if anything had happened. I definitely think it would have helped, because you’re thinking your emotions are one thing for one reason, and they’re not. And they’re not getting dealt with, and it needs to be dealt with.” (LoGiudice and Beck p.477)  “I never had anyone ask me about my past abuse.” (LoGiudice and Beck 2016 p. 477)  “Never, never did anyone ask.” (LoGiudice and Beck 2016 p. 477)  “I started opening up about when I was – what happened to me when I was five… but then I felt like I should have never opened up… cause it’s like I don’t want people to feel bad for me.” (Participant 14) (Sobel *et al*. 2018)  “I would like to see MCHN set aside more time, not just for the baby, but for the mum as well. To help them through that transition process of accepting the changes and moving on, but also addressing any other issues that they may have, because everyone is so different. I might have come from an abused childhood, but there are also women from terror and war-stricken countries and stuff like that. They’ve been abused too, in different ways. I thought the whole maternal health nurse thing is one of the best ideas that the government has in place and its real value for money. But the system needs to help more, it needs to help the parents through that transition process; I think that’s what’s lacking.” (Caitlyn) (Coles and Jones 2009)  “Some people communicated with each other, and some people came in like they just didn’t know anything or did not take the time to read what was documented.” (Participant 7) (Sobel *et al*. 2018)  “I really don’t have time to open this can of worms. It takes long enough to get through a perinatal visit without taking on the job of social worker.” (Midwife) (Rhodes *et al*. 1994)  “What’s the matter with you then? Have you been abused or something?” (Kitzinger, 1997) |
| **Activating Events**  **Four Sub-themes: Positions in labour; Intimate Procedures; Communications with Healthcare Professionals; and Loss of control** | “I was watching for things that were happening to my body and that took my mind off it (labour). You know, it was like things were not just being done to me. That was a real help to me” (Rhodes *et al.* 1994)  “During this labour, the nurse didn’t know I was doing anything, and we were pretty classic according to my handbook. Of course, I was adamant enough not to let them do anything at that point, I knew what I was doing” (Rhodes *et al.* 1994)  “I thought she was weird. It took 20 minutes for her to let me do one vaginal exam. She told me when I could check and how I had to touch her leg first.” (Rhodes *et al.* 1994)  “When it came right down to it there was no way that I was willing to put myself in the situation where anyone would have that kind of control over me … I had my baby at home with a midwife. I made elaborate preparations and back up plans.” (Rhodes *et al.* 1994)  “It’s sort of like, when you’re being raped and bashed and you’ve got no control over what’s happening and it’s the same with giving birth, you can’t really control it, the contractions keep coming…” (Montgomery, 2013 p.31)  “I knew I was going to have to deliver this baby … and I knew there were going to be more examinations and things being taken out of my control again, because I didn’t feel strong enough to say ‘No, I don’t (you know) I want to do this” (Montgomery, 2013 p 180)  “I tried to hang onto control up until the point where I was going into the c-section thing, and then I gave up pretending to have control and that was fine. It felt awful, but it felt better than pretending. That was the point where I really started screaming. They wheeled me down the hall, screaming my head off” (Montgomery, 2013 p. 255)  “I had a birth plan, which was a part of my trying to have control, although knowing if there was like an emergency… you know, that was a different story.” (Participant 19) (Rhodes *et al*. 1994)  “I felt totally disempowered. When I walked out, I was crying uncontrollably and yelling at the children. All these things that are chain reaction to… her making me feel bad. She made me feel bad about myself as a mother, and about my children and their behaviour. I think, as a patient, you have to start exploring different areas to actually connect them with your past abuse. Sometimes you do things you don’t realise you do because of your past. Its just the way you are used to dealing with people. Like the doctors and the health nurse, I felt like I couldn’t really say too much about it because I couldn’t just stand up and say no.” (Alice) (Coles and Jones 2009)  “Just more watching people change her, being more curious, and just wondering what people are thinking when they were changing her and what are they going to do – just not letting them change her away from me either – I like to hover.” (Liz) (Coles and Jones 2009)  “I think ‘Should you be doing that?’ it’s just doubt, and concern about how appropriate it is. I knew what they (doctors and MCHN’s) were doing, but no one ever said, ‘I am going to do this now’. It’s something that’s got to be done… but if it got too bad… I don’t know (what I would do).” (Liz) (Coles and Jones 2009)  “I would be very uncomfortable with a male doing an examination, simply because I was abused by a male and I don’t trust any of them, especially in the 30-to-40-year-old age group. If I had to hand her over for surgery, it would be incredibly traumatising, I mean it’d be bad enough being a mother and having to hand her child over for surgery but having been a child who got abused and having my trust violated, it would be extra hard… to hand over another innocent child and trust… I trust you not to hurt her… especially if it was a male in that category of men I distrust… it is just difficult.” (Penny) (Coles and Jones 2009)  “I turned around and chose a woman doctor, and it must be because having the man obstetrician bothered me… I think I chose a female doctor and then a midwife around the fourth month because it felt more comfortable to me.” (LoGiudice and Beck 2016 p.478)  “And then with breastfeeding, there was no way, I couldn’t do breastfeeding. I felt that it was dirty. My cousins, some of them and my friends, they breastfed, and I don’t know how they do it, because to me that’s not what it (the breast) is for… I know it’s what it’s for in my head. I know it is, but I just feel like it’s a dirty thing.” (LoGiudice and Beck 2016 p.478)  “I couldn’t just like just walk away from the situation… I just kept on feeling like I was trapped with him (son) (…) I just felt like I’m going to be really really vulnerable, um, and kept having this image of me like on the bed, you know I can’t get out that situation I can’t… I have no control over it.” (Evelyn) (Byrne *et al*. 2017)  “We got to the pushing stage and she has never able to put the effort into it. She seemed afraid. She’d start talking about ‘I can’t stand the pressure. I can’t stand the feelings down there… I can’t do it. I can’t do it!’ Pushing was through the mouth and nose breathing – some screaming and just pulling back. I think the entire second stage was through the contraction effort and no through hers (…) She would look like she was doing that (holding her breath and push down) … but it wasn’t really going anywhere. It was odd because she would make the right noises, she would even make grunting noises but when I’d look up or put my hand on her belly there wasn’t any effort, wasn’t anything going on there.” (Rhodes *et al*. 1994) |
| **Sub-themes: Positions in labour and Intimate Procedures** | “I don’t remember talking to anyone about how I felt because I didn’t know myself; I was just empty. I sat at home when I had stopped working. I (sat) for hours in a chair, with no music and nothing, and just looked out into the air. I just didn’t feel like doing anything.” (Jonsdottir *et al*. 2020 p.679)  “I just felt like I needed to vomit. I was in a state of shock. This is such a sensitive area, and the body remembers the touch that happened when I was a little girl. I really felt the body in this examination.” (Jonsdottir *et al*. 2020 p. 679)  “And it just brings you back to when you were touched there before.” (LoGiudice and Beck 2016 p. 477)  “And then of course it gets more invasive as you’re further along in your pregnancy because they check to see how much you’ve dilated… 2 fingers, 3 fingers, 4 fingers, and that kind of bothered me, that actually did, the verbal, because the nurse was always there and of course she was writing down everything he (the doctor) was saying and for some reason mentioning how many fingers, that triggered, that was like a trigger.” (LoGiudice and Beck 2016 p.477)  “this squirmy, slimy thing (baby).” (LoGiudice and Beck 2016 p. 478)  “… telling me what I had to do and that I couldn’t move. I felt trapped is what it was. Just like the trapped little girl that I was.” (LoGiudice and Beck 2016 p. 478)  “I think I was very compartmentalised, and I don’t remember it (childbearing) being traumatised. It definitely was very compartmentalised.” (LoGiudice and Beck 2016 p. 478)  “I didn’t feel like I was heard. I didn’t feel like I was respected. I wasn’t informed.” (LoGiudice and Beck 2016 p. 478)  “I remember falling asleep. I fell asleep. And then they came into the room, and it was sort of a rush. Okay, let’s get this baby going, let’s get this baby out. Sort of scary, because the nurses walking in, the doctors walking in, and everyone just sort of walking in, like okay it’s time to go. It’s like, oh, where are we going?” (LoGiudice and Beck 2016 p. 478)  “Memories are fragmentary and disconnected: A picture without a story, a story without a picture, feelings without pictures or stories, often they are preceded by an aura: … and then the memory comes.” (Rhodes *et al*. 1994 p.78)  “Looking back on it today, I see I didn’t remember the abuse until I was 36. I was 18 then (in labour), and I had blocked it out for two years. I had forgotten it, so it was completely blocked.” (Rhodes *et al*. 1994)  “This woman panicked in very early labour, cried loudly with each contraction, and pressed her legs tightly together. She clung to her husband’s hand and cried “Help me! No, I don’t want to do this.’ When asked ‘Has someone hurt you before where I am touching? Has anyone before ever touched you sexually without your permission?’, She denied abuse. However, her husband confirmed privately, to the nurses that he had long believed that his wife’s father had molested her. He had feared ‘putting her through that’ and had not discussed it with her” (Rhodes *et al*. 1994)  “The body not only seeks truth, but also, for want of a better word, it stores truth. When we’re ready, our body may provide us with clues about painful truths that our conscious mind has repressed. Many of is receive the precious gift of memory through the body first.” (Rhodes *et al*. 1994 p.193)  “I was spread-eagled on a bed, my arms tied to drips (intra-venous lines), someone fiddling around down there – it brought back the bondage.” (Rhodes *et al*. 1994 p.39)  “The memory of the violations during my childhood was locked in my birthing muscles for all these years, only recently coming to the surface of my conscious awareness. Such armouring can hinder the ability to open one’s body, to trust that the body really can work okay and be safe. It is hard to believe that the intense sensations in that region of the body do not have to mean bad things are going to happen.” (Rhodes *et al*. 1994 p.216)  “My body is my enemy because it is the one who let it happen, and my mind is my ally. And that it (my body) was helpless, I couldn’t know then, and who allies with the helpless anyhow?” (Rhodes *et al*. 1994 p.41)  “These women are off the head of the bed by the time you’ve got your hand at their ankles, and you’re sure the circulation in your hand has been cut off. No matter how gently or how much of a trust relationship you feel like you’ve built, the minute you start touching them there, they’re gone.” (Rhodes *et al*. 1994)  “She pushed backwards. She put all her effort into her chest and arms instead of directing her energy down and out. She pushed on her arms like she was putting on brake. The frustrating thing was she did such extensive seesawing of the baby. She was a gravida there and could have pushed that little baby out in just a few pushes. What she did was to push her down to the point of starting to crown with the contraction. We could see about 6 centimetres worth of the baby’s head, but then she tightened her vagina and buttocks and sucked the baby way back up so that she started from the same place each time she pushed.” (Rhodes *et al*. 1994)  “The birth terrified me. I thought I was going to die. I felt like that during the sexual abuse. When you get in a situation. Where you feel like you’ve been violated or you feel like your life is in danger, or someone has so much power over you they can do anything, you’re out of control… That’s terrifying and that’s the feeling I remember when I was giving birth. I was going to die and there were no two ways about it.” (Rhodes *et al*. 1994)  “(Pushing) felt like somebody that was over a thousand pounds was sitting on top of me.” (Rhodes *et al*. 1994)  “’I’m gonna check you, and you better not move or I’m going to be forceful’ when she said that, it really hurt me.” (Participant 5) (Sobel *et al*. 2018)  “They were just saying really positive things saying, ‘you can do it’. And you know, they didn’t use negative words. So, it definitely helped me feel encouraged.” (Participant 5) (Sobel *et al*. 2018)  “(It’s) been a long time, but still when someone says rape, it’s a trigger. I still think of these things.” (Sobel *et al*. 2018)  “I just didn’t want to be exposed. It was more that I didn’t… I was uncomfortable that I was naked. Of course, I’m having a baby, you’re naked. I brought like brand new thigh highs… I thought I could like cover up a little bit.” (Participant 20) (Sobel *et al*. 2018)  “I was always jumpy when someone would open the door. Cause I didn’t want the wrong person to come visit me (at the wrong time).” (Participant 14) (Sobel *et al*. 2018)  “During surgery they kept me as covered as possible. I mean like even my arms. I was like ‘no, cover me. My legs, my feet, everything. I want everything under that blue mat.’. You know, besides what they had to see. I was very, very nervous about it prior to that.” (Participant 20) (Sobel *et al.* 2018)  “They never asked me if I wanted him (partner who was her abuser) in the room when I was getting examined. It would have been nice if somebody would have been like… ‘Do you want him to be here?’ Nobody asked me.” (Participant 2) (Sobel *et al*. 2018)  “In active labour she assumed a childlike voice, she threw her head up to the ceiling, clearly she was not in the same room with the rest of us in her mind, and (she) pleaded quietly but hysterically: ‘Don’t hurt me there. If you’ll stop hurting me, I’ll be good. I promise I won’t tell anybody!’” (Montogomery, 2013 p. 218)  “My labour set off a whole series of flashbacks. For which I had no context. I was still in denial about my abuse. I seriously thought I had gone to hell.’ (Montgomery, 2013 p.133)  “I do still have moments when I’m caught unaware, smells I’m particularly sensitive to, if I smell something that reminds me of being back there.” (Montgomery, 2013 p.45)  “It’s pretty spooky because the first thing that happens is, I lose eye contact, I can’t communicate verbally, I just completely shut down… the worst event is total paralysis. I shake uncontrollably and then I’m paralysed. I don’t have any control over my whole body. My whole body is paralysed; I can hear, but I can’t talk.” (Montgomery, 2013 p.233)  “I think the bottom half of my body was gone somewhere else… and the nurses were just, you know, ‘this woman is marvellous! She doesn’t feel pain…’” (Montgomery, 2013 p.256)  “I have enormous resistance to opening my legs on demand.” (Sophie) (Kitzinger, 1997)  “Of course I hate them but I just detach my mind from my body. I’ve had years of practice doing that – with my Stepfather!’ (Anonymous) (Kitzinger, 1997)  “I can see myself walking out of the hospital gate feeling guilty, not a good wife, dirty, in pain, humiliated. They were holding me down while the doctor tried to take a smear; they were shouting at me. It was painful. I just wanted to get away. They said my marriage wouldn’t last and I should be ashamed of myself for carrying on like that. When I started to struggle they should not have held me down. It does not seem a big thing but the feelings are still there.” (Morag) (Kitzinger, 1997)  “I was on my back where I don’t like to be, and I was out of control, and I was in pain.” (Anonymous) (Kitzinger, 1997)  “(Childbirth was) like being sexually abused all over again.” (Anonymous) (Kitzinger, 1997)  “The same indignities, lack of control, humiliation, and depersonalisations. My despair led to a total loss of self-esteem and I attempted suicide… My horror of hospitals and doctors has increased. I find myself terrified at the thought of an examination.” (Anonymous) (Kitzinger, 1997)  “(I felt) nothing. I was just absolutely exhausted. I had nothing left, and there was just some baby there on my stomach. I was just a complete wreck.” (Jonsdottir *et al*. 2020 p.679)  “I felt like I was being abused all over again and I started to get really really upset and they just said it’s okay and it’s just your hormones.” (Ella) (Byrne *et al*. 2017)  “I no longer knew I was giving birth. It was very unreal, but so is a rape. I felt that in a way, I left my body, like when I was raped. I did not know where I was, if I was above myself looking down. But it felt very similar.” (Participant 4) (Halvorsen *et al*. 2013)  “Looked down and saw myself from above, like a slaughtered animal lying there that they could do whatever they want to. Could not move a muscle, and real scared. I am lying there stunned and cannot get away. Cannot take in what is happening I am good at leaving the crime scene.” (Participant 7) (Halvorsen *et al*. 2013)  “Males are a trigger, but the main trigger is lying flat on my back with my underwear off.” (Lenora) (Coles and Jones 2009)  “Everything really went wrong. On the second day after birth, a midwife comes to me and asks, ‘Are you okay to pee?’. And then suddenly, it dawns on me: I had urinary incontinence without realising it. Of course, I have never really recovered from this (CSA and birth trauma) (Jonsdottir *et al*. 2020 p. 679)  “The person I think of is the 15-year-old kid who gets on the table and flops her legs open, and seems like she hardly knows she’s doing anything. You expect that with a woman who has had 5 kids but not with a 15-year-old. These are the people who labour quietly. I remember a woman with a history of multiple sexual abuse(s). When her daughter was born she had an extremely long labour but nothing bothered her. She remembers the details of her birth and how goof people said she was at it. She didn’t have any anaesthesia to account for it.” (Rhodes *et al*. 1994)  “She laboured and had the baby but was completely spaced out through it. She just never said anything through the whole labour. She was just one of those people who you think are wonderful in labour except they weren’t there.” (Rhodes *et al*. 1994)  “That was probably the worst pain up to that point that I had experienced but it was not the first time that I had felt that way and I just sort of went away mentally. I think that’s why a lot of things were a haze. I just blocked everything all around.” (Rhodes *et al*. 1994)  “It was extremely hard, a lot of just terror and I just felt such a big part of the abuse when I was having her. It was just like being surrounded by it for hours and hours and hours and everybody said I was just like a little girl, I wasn’t myself anymore… I became somebody else like a crying child shut up in a closet or something… they didn’t recognise me.” (Rhodes *et al*. 1994) |
| **Sub theme: Loss of Control** | “I did not want to be on my back with my legs up, but they hold my legs. Something happened to me around that, being held in place. For me, it led to just giving up, they could do whatever they wanted. It was kind of… all the way up to that point, I was protesting.” (Participant 9) (Halvorsen *et al*. 2013)  “When they laid me on the operating table I felt as if I died. My whole body disappeared. I felt nothing, I was gone, I had no way to get away. I could not get away.” (Participant 10) (Halvorsen *et al*. 2013)  “When they put in that ‘vacuum cup’ it was the first time I really had thought about that rape in years. I was back in it, being held down and not being able to move. In any case I was completely naked. I felt a kind of shame too. Up to this point I was kind of angry, in a way. But then it was a little bit like when ‘he’ (the rapist) – it was kind of too late. Nothing left to fight for. Afterwards I felt I’d done such a terribly bad job.” (Participant 2) (Halvorsen *et al*. 2013)  “Just lying there on your back and you’re in the same position when you again go into the same state as when you were raped, plain and simple. You’re lying there and things happen down there (points between her legs), the feeling of being held down. It was really strange… my body held back and would not do it. I wanted to give birth but my body would not do it.” (Participant 1) (Halvorsen *et al*. 2013)  “I was stuck in the bed, they could do what they wanted with me. They were saying something about them seeing that I am in pain, but ‘they will be quick’. They pull away the duvet I am clinging to for dear life and they pull up my top. Lying in bed, vulnerable, I leave the room; they can just do whatever they have to.” (Participant 10) (Halvorsen *et al*. 2013) |
| **Sub-theme: Role of the Healthcare Professional** | “I opened up to (my midwife) because I felt comfortable with her. That’s it.” (Participant 14) (Sobel *et al*. 2018)  “I just didn’t… I stopped. You know, I’m someone who can advocate for myself pretty well, but I just kind of felt like I didn’t have the space to really have the conversation.” (Participant 19) (Sobel *et al*. 2018)  “(Disclosure) affected it in a good way because they already know what they had to do and what they had to be careful of and look out for and all that stuff.” (Participant 17) (Sobel *et al*. 2018)  “They need to know, you know what I mean? We have a chart for a reason. It says on the chart either what we’ve been through, some of our history – because of the fact that you know they need to know.” (Participant 1) (Sobel *et al*. 2018)  “And I feel like of course you don’t want them to know everything, cause it’s private. But if there was something in the patient chart with the consent to let them know: okay, this person has had trauma before – I feel like they would treat it in a different way and be sensitive to their needs.” (Participant 5) (Sobel *et al*. 2018)  “I think they could have communicated with each other more, especially with my primary obstetrician, they could have communicated with him and found the history or something and talked to him about what’s going on at least. I’m sure he could have given them a little run down of my history. That way, they know.” (Participant 2) (Sobel *et al*. 2018)  “It seemed like every time a new nurse or new midwife came in, they didn’t know nothing about the birth plan. I was like ‘Oh it’s in the chart’. And they were like, ‘oh we will check it’. So they don’t really check your chart before they come see you.” (Participant 7) (Sobel *et al*. 2018)  “My primary doctor, he was just like, ‘What do you want? Do you want to do C-Section? Do you want to do vaginal delivery? You let me know and we’ll go that route as long as it is safe… That’s what I wanted to hear.” (Participant 2) (Sobel *et al*. 2018)  “‘Thank God that I had a C-Section’ I wanted that C-Section with him. It’s a hard birth. That’s the best option for me. I would rather go through that.” (Participant 10) (Sobel *et al*. 2018)  “Being that the information was in the computer, I thought they would be a little more sensitive.” (Participant 5) (Rhodes *et al* 1994)  “I never came forward because I thought they were going to take my baby away.” (Byrne *et al*. 2017)  “When she gave me the initial, you know, the history form… when I saw ‘were you abused?’ I said no. There was no way I was going to tell her.” (Montogomery, 2013 p. 366)  “If she (the RN) would have perhaps said… offered an opening some little opening like ‘How is this on you? Sometimes this (childbirth) brings up a lot from people’s past’, or some little sense of awareness that it could be an issue, I think I would have right away given it… a teeny little opening would have taken it. but I never had the sense that there was one.” (Montogomery, 2013 p. 253)  “I was just ashamed of myself … I was just asking if I could be heard from the other rooms because I was so loud. It was just awful. I never screamed so much.” (Jonsdottir *et al.* 2020 p.678)  “I felt so overwhelmingly responsible, and I was extremely careful and did everything really well, but I was terrified!” (Jonsdottir *et al*. 2020 p. 679) |
| **Sub-theme: Emotional response** | “(I) found out pretty early on that it was a girl, and my god, how relieved I was! I felt like I was just having lollipop and lace inside of me. That birth went brilliantly as did bonding and breastfeeding.” (Jonsdottir *et al*. 2020 p. 680)  “I actually enjoyed it.” (LoGiudice and Beck 2016 p.477)  “It’s just a miracle, just the fact that something’s growing and living inside of me.” (LoGiudice and Beck 2016 p.477)  “I was actually happy to have my baby, I didn’t have any negative feelings about it.” (LoGiudice and Beck 2016 p. 477)  “And I think in the back of my mind I felt that I did something wrong then, when I was abused, and then now when I got pregnant.” (LoGiudice and Beck 2016 p. 477)  “I’m also a registered nurse, so I’ve gone through nursing school. I’ve been in the delivery room and I’ve seen it is a very beautiful thing, but I’m like this (mine) was not. There are births that I attended when I was a nursing student, the mother was listened to, the lights were turned down if she wanted. The nurse would listen: you only want the father in the room? Do you want students in the room? She (the patient) was covered. What happened (for my birth)?” (LoGiudice and Beck 2016 p. 477)  “The entire time I was pregnant I didn’t know what I was going to have because I wanted to be surprised, but I had it in my head that I was going to have a boy, because I was so afraid of what would happen if I had a little girl.” (LoGiudice and Beck 2016 p. 478)  “There was the protectiveness that you feel of your children afterwards which is unique.” (LoGiudice and Beck 2016 p. 478)  “I’m quite a late mum really at 37 when I fell pregnant and um purely well to do with the sexual abuse and to do with um having no confidence around children.” (Byrne *et al*. 2017)  “I’d never had even crossed my mind at that point that I’d ever become a mother I just thought my my mental health problems were too strong… I would be no good as a mother.” (Byrne *et al*. 2017)  “I felt like I was being judged quite a bit and um… I’d lost my identity.” (Byrne *et al*. 2017)  “I kind of, I don’t think I accepted like I literally like within the last couple of months I’ve only just acceped my Mum role because I think. I didn’t, for me in order for me to accept it means that I’ve… it’s change isn’t it like a big change, um and I kept kicking against it.” (Byrne *et al*. 2017)  “It was very different to become this normal person going to normal hospital appointments, um, that normal people would do I know this is all very normal but um, it was quite a nice experience thinking I’m doing things that um people do without mental health problems (…) I was moving on with my life and leaving the mental health problems and becoming not a normal person as such but becoming um something that normal people do (…) I wanted to be normal but I didn’t know how to.” (Dolly) (Byrne *et al*. 2017)  “Um I was quite naïve in the fact that um… I know it sounds really silly not but um, I wasn’t quite sure what happened when you got pregnant.” (Ella) (Byrne *et al*. 2017)  “I think I wanted to get pregnant because um… I want my abuse to leave me alone, so um and I wanted… I just wanted to feel safe (…) I thought I’m going to have to be pregnant all the time um to keep him away from me because that’s the only way.” (Byrne *et al*. 2017)  “I’ve always felt um… uncomfortable… anyway, um, like I just for a long time I just wanted to like get out of my body like I just kept kind of envisaging like having a zip down the back of my back and stepping out of my body because I just, hated it like and it’s it’s feeling trapped isn’t it in your body you can’t really help it (…) I just don’t ever I didn’t ever feel… really that… safe and comfortable in myself.” (Byrne *et al*. 2017)  “I wouldn’t be without any of them now and they’re an absolute god send to me they keep me alive.” (Ella) (Byrne *et al*. 2017)  “It is a life changing experience you know, having a baby really changes your life. Luckily for me it has done for the positive um… it was a journey, a good one. I wouldn’t change it for the world.” (Dolly) (Byrne *et al*. 2017)  “My doctor didn’t want to know he just said to me one day that you’re very young very in-experienced mother go home and read some books, and that’s what I got from my doctor.” (Ella) (Byrne *et al*. 2017)  “You think no-one’s going to believe you.” (Ella) (Byrne *et al*. 2017)  “I just don’t ever I didn’t ever feel… really that… safe and comfortable in myself.” (Byrne *et al*. 2017)  “I found it hard to sleep with him to get pregnant. I found that quite, um, traumatic.” (Dolly) (Byrne *et al*. 2017)  “I found it hard to sleep with him to get pregnancy I fount that quite um traumatic and the thoughts of it start with I was sort of going through how I could get pregnant without having to have sex… I sort of forced, forced myself in the end thinking that this is the only way.” (Byrne *et al*. 2017)  “I wouldn’t want to look after a boy in case he turned out to be some sort of abuser or um anything like that or just because of their male bits I wouldn’t want to change male bits and things like that and to have that put in my face all the time of a, to see, their willy and stuff like that.” (Byrne *et al*. 2017)  “Pregnancy has been a big turning point in how I saw my body.” (Dolly) (Byrne *et al*. 2017)  “I thought I’m going to have to be pregnant all the time, um, to help him away from me because that’s the only way.” (Ella) (Byrne *et al*. 2017)  “I’m never going to get this chance to… rectify like what’s happened to me.” (Dolly) (Byrne *et al*. 2017)  “I always resented him (son) in in in some way and now I, I find that very hard, um, to think that I resented him.” (Ella) (Byrne *et al*. 2017)  Yeah, it was a lot of people… too many people around. I didn’t feel like… in my head I didn’t feel like it was necessary to have that many people.” (Participant 1) (Sobel *et al*. 2018)  “I was so concerned with being covered up… I would have been devastated (by a vaginal delivery): I did not know how I was going to keep my clothes on and have a baby.” (Participant 20) (Sobel *et al*. 2018)  “It was a journey, a good one, I wouldn’t change it for the world.” (Dolly) (Byrne *et al*. 2017)  “My mum didn’t know my dad would abuse me when she married him; how do I know my husband won’t turn out like that?” (Kitzinger, 1997)  “I didn’t like to touch his bits because I thought if I did I’d end up like my dad and do it to him.” (Kitzinger, 1997)  “’Oh my God!’ It’s a girl. I can’t bear it if she has to go through what I’ve been through.” (Kitzinger, 1997)  “If he wanted a cuddle, you don’t take it as he (son) wants sex. I think it’s helped having him, to see what a seven-year-old looked like!” (Kitzinger, 1997)  “I’d think ‘so what, you are feeling pain, it doesn’t matter; you are depressed, so what?’ Before I had any children I could live quite happily with the fact. ‘So, that happened, it doesn’t matter, it’s a secret’. But when I had children… I knew I had to do something about it.” (Amy) (Kitzinger, 1997)  “I recall the birth as dark and ominous, a big black hole I was afraid to fall into, because there he stood, the man who raped me and he was grinning this awful grin. I know I fought not to fall down the hole, because if that happened I would lose my mind. It was vital to stay on the edge and not fall in. My husband realised I was struggling in my own world, fighting with someone outside the room. It cannot have been easy to be him while I was battling another man in the midst of the birth of our child.” (Participant 3) (Halvorsen *et al*. 2013)  “I was alone against them (their emotions and needs). All the information was given with their hands me… I tried to tell myself ‘Relax! Get a grip!’ but it was no use.” (Participant 10) (Halvorsen *et al*. 2013)  “I felt he was brutal, it was just kind of a ‘whoosh’… felt he just broke my legs apart, and right in and just go ahead and check me. It was such a helpless situation to be lying there… in way like… a bit of a violation really. Even though I tried to tell myself, sort of sensibly that ‘they do have to do this’ and ‘I guess they need to check that everything is ok’” (Participant 4) (Halvorsen *et al*. 2013)  “That the midwife did not talk to me, did not address me – and that I was not allowed to be involved. I was just a kind of ‘robot machine body’ that was there to give birth to a baby – where nobody saw ‘me’. There is something very degrading about being treated like a birth machine that is just something to be repaired. You are not a machine that is going to give birth to a kid, you are there as a person too. You are not just a body that another body is going to come out of. So in a way it is ‘a body’ that gave birth – but it was not me.” (Participant 2) (Halvorsen *et al*. 2013)  “It was sort of the baby it was all about, not ‘me’ at all. It was so strange really – as if I was not even there – I was not there. I was just – I was not even a patient, really – was actually nobody. There was just a baby who was going to come out of me.” (Participant 4) (Halvorsen *et al*. 2013)  “(I) recall that I felt dirty! I felt violated, and I really wanted to brush my teeth. I felt I wanted to brush away something or other: and this has something to do with me feeling really dirty – and those old nightmares about the hands came back.” (Participant 1) (Halvorsen *et al*. 2013)  “It got so important to get on my feet again, after the caesarean section. Everything had to be washed away, sweat, blood, filth, bits of tape, and most important, hands. All the hands that had been there, had to go.” (Participant 10) (Halvorsen e*t al*. 2013)  “I felt just gross. They shoved me further and further away from myself, just slammed on. They stood there all three with their hands in me, down there. Was not that nobody saw me or talked to me. I was just empty. I hate my body thinking of myself as one thing, my body as something else, and we are not working together. My body is just gross.” (Participant 10) (Halvorsen *et al*. 2013)  “well, it feels like certain of your body parts are not yours no more. It is just something someone take all away from you. You sit there or lie there, just like that, and just ARE like a carcass or a beached whale in my case. I felt like I was just laying there, stuck, and could not come back to my own self. I feel I’m still lying there, when I ought to be lying out to sea, swimming.” (Participant 9) (Halvorsen *et al*. 2013)  “scared if he was OK or not.” (Records and Rice 2002)  “I was afraid of surgery.” (Records and Rice 2002)  “It was the needle (epidural).” (Records and Rice 2002)  “I wasn’t ready to be here yet – worried a lot about my job and whether to pay a baby sitter all the little things and I can’t just walk out the door by myself, now I have to pack the whole house and now I have somebody too worry about for the rest of my life.” (Records and Rice 2002)  “I worry because I don’t want her to get sick at my breast.” (Records and Rice 2002)  “I would start crying when I looked at her because I didn’t get the feeling. I felt bad.” (Records and Rice 2002)  “I was sad and crying.” (Records and Rice 2002)  “To be surrounded in the depression.” (Records and Rice 2002)  “I was distanced from my family.” (Records and Rice 2002)  “I didn’t spend too much attention in the beginning (on the baby) because I was out of it.” (Records and Rice 2002)  “I was panicking.” (Records and Rice 2002)  “The pain was out of control.” (Records and Rice 2002)  “A million things pass through my head.” (Records and Rice 2002)  “Just seeing everyone’s expression, I knew something was wrong.” (Records and Rice 2002)  “I took my baby to a neighbour and he was crying and I didn’t know what to do.” (Records and Rice 2002)  “I was so nervous…. I didn’t know what to do.” (Records and Rice 2002)  “I was always the defensive.” (Records and Rice 2002)  “They told me ‘you’re gonna feel a little cramp’ and I felt like a heap of people had kicked me.” (Records and Rice 2002)  “Something was lacking in the medication.” (Records and Rice 2002)  “The pain was terrible all the time.” (Records and Rice 2002)  “Can’t push.” (Records and Rice 2002)  “She was turned around and then, go back and turn her around, and then the umbilical cord was wrapped around her.” (Records and Rice 2002)  “His heart rate went down.” (Records and Rice 2002)  “My baby was too big.” (Records and Rice 2002)  “When I am alone in the room, I’m looking for signs… I think because my GP is a male, and of course, he has to touch me in very private places.” (Montgomery, 2013 p. 232)  “One of the nurses grabbed my breast and shoved it in my daughters’ mouth… I was horrified.” (Montgomery, 2013 p. 155)  “The first thing I felt when they put him on my stomach, I thought to myself, I feel clean, you know like all that bad stuff was washed away.” (Montgomery, 2013 p. 33)  “I’ve certainly never liked my body but once I’d had, you know, it had sort of served me well after these two births and I thought, ‘God… actually, it’s a pretty good body.’” (Montgomery, 2013 p. 178)  “The postpartum is more painful than the delivery when the baby got down there… (you) feel more with your first child.” (Theresa) (Records and Rice 2002)  “I panicked because I was in such pain with my first one, so I thought everything was gonna go smooth (this time) but then it started hitting my butt bone and was coming faster and faster and I panicked.” (Maria) (Records and Rice 2002)  “I felt fear… because one doesn’t know what you have to do, how to educate, how they get when they are sick,… like it’s an encounter of ideals in one’s head of that love… one has to… to get depressed a little. When I get depressed, I get very depressed. I tried to tranquilise that feeling because if not, I would be in a very bad place.” (Michelle) (Records and Rice 2002)  “I’d never had even crossed my mind at that point that I’d ever become a mother, I just thought my mental health problems were too strong… I would be no good as a mother.” (Dolly) (Byrne *et al*. 2017)  “I was moving on with my life and leaving the mental health problems and becoming not a normal person as such but becoming um something that normal people do.” (Dolly) (Byrne *et al*. 2017)  “You think that no-one’s going to believe you (…) I never came forward because I thought they were going to take my baby away… I would have felt like he’d won (…) I was never asked (about the abuse), never, not when I took the overdose not when I had the children… never asked.” (Byrne *et al*. 2017)  “I always resented him (son) in in some way and now, I find that very hard um to think that I resented him (…_ I carry a lot of guilt about the way that um I, I dealt with the children and looked after the children when they were younger all because of him, he (abuser) took so much of my life and my time my previous time with my children… I feel a hell of a lot of guilt.” (Byrne *et al*. 2017)  “It was quite um a shock (having a boy) I was, it took me a while to get over it… like weeks to get over it because I’m never going to ger a chance to… rectify like what’s happened to me.” (Evelyn) (Byrne *et al*. 2017)  “I imagine a lot of people who have suffered sexual abuse might think they don’t want a boy because um they could possibly be an abuser although I didn’t want a girl for the reason that I didn’t want her to be abused because I knew I’d love this child and I wouldn’t want any harm to come.” (Dolly) (Byrne *et al*. 2017) |
| **Sub-theme: Intimate Procedures** | “I had to have um… um… an internal examination… when I had cos I had to be induced so I had to have a pessary… that is quite traumatic to me even like I remember I’m sure it wasn’t as bad as I remember but it was pretty horrible… I just found it a really really horrible, um, experience.” (Evelyn (Byrne *et al*. 2017)  “It’s not like you’re just getting an eye examination. It’s a little more of an intense process both physically and emotionally.” (Participant 5) (Rhodes *et al*. 1994)  “I always have a slight flashback, but I think as I’m getting older it’s becoming less and less in terms of the sexual abuse.” (Participant 4) (Rhodes *et al*. 1994)  “Yeah they explained. They did (but) it was a different doctor or midwife every time checking me. And so that was uncomfortable.” (Participant 7) (Sobel *et al*. 2018)  “It’s just 20,000 things running through my head at the same time and it’s just not comfortable for me at all.” (Participant 2) (Sobel *et al*. 2018)  “I was only checked once during my prenatal. So that wasn’t bad. And maybe it’s because she knew my history and was trying to be sensitive. And she asked. It wasn’t something that was forced on me.” (Sobel *et al*. 2018)  “Examination is pretty uncomfortable and having myself physically examined during pregnancy… Even before I was pregnant, pap smears and stuff were incredibly uncomfortable… it would be way more comfortable setting fire to my arm.” (Penny) (Coles and Jones 2009)  “I just felt really violated because they were all coming in and feeling what my cervix was doing and 1 minute it was fine and then the next one would come in. it was just in and out, like a dunny (toilet) door half the time… and it hurt, like… I just wanted to say, I was emotional as well, ‘Oh god, just leave me alone!’. I had to bite my tongue and do whatever. I think I had something like six internals within 10 hours. It would have been all right if just one person had done it, but there were three or four different people. I think I just felt uncomfortable and a bit violated. Whenever you feel violated, that becomes the number one feeling. As a young girl I didn’t understand when it was happening, but as an older woman I understood.” (Caitlyn) (Coles and Jones 2009)  “I felt like because there was a purpose to it so like, you know, I was okay with it.” (Participant 19) (Sobel *et al*. 2018) |
| **Sub-theme: Communications with Healthcare Professionals** | “He was absolutely shocking, this doctor. He just didn’t listen. I told him I did a self-examination and I didn’t need him to do a breast examination, and he said, ‘That’s what we do’ so he did it anyway. I had only just had a pap smear 6 weeks before that, and he insisted on doing a pap smear and a pelvic exam at the same time. I said to him ‘I’ve just had a pap smear, I don’t need one’, ‘Oh no, we do one when you are pregnant and you have to do one at the start of your pregnancy’. So, he was just totally horrible. If I had known what I know now, I would have just got up and walked out and said ‘you don’t need to check me for any of those things. What are you – a pervert? Go away!” (Alice) (Coles and Jones 2009)  “I guess it makes me feel angry, because people are using their profession as a way of putting you down and making themselves feel better. You feel guilty about not sticking up for yourself. Not having the right words to say. I think that professionals get the same kick out of power over others as abusers. Abuse only occurs because someone feels they have power and they can dominate another person.” (Alice) (Coles and Jones 2009)  “When I am alone in the room, I’m looking for signs. I think because my GP is a male, and of course, he has to touch me in very private places. That was hard; at the very start I was looking for signs, like when I was on the bed (examination couch), maybe his hip would touch my arm or his tummy would touch my arm and I would go… oh… what are you doing? Then I would realise now he’s not doing it abusively… he’s just trying to do this job. Things like that still affect you strongly, so you know that you’re on edge worrying. I don’t think we would ever get over it. I think we just need to learn how to live with it and that’s it.” (Jane) (Coles and Jones 2009)  “There was one bad experience during the IVF treatments when one of the nurses rushed me. Normally I keep control of the speed of vaginal examinations and tell them to stop. One of the nurses didn’t hear me, and I went into trauma response. It’s pretty spooky because the first thing that happens is, I lose eye contact, I can’t communicate verbally, I just completely shut down… the worst extent is total paralysis. I shake uncontrollably and then I am paralysed, I don’t have any control over my whole body. My whole body is paralysed; I can hear, but I can’t talk.” (Lenora) (Coles and Jones 2009)  “I think for some people avoiding language their perpetrators might have said them. Um because the language of labour and delivery is a lot like ‘relax, you’re almost there’, like ‘you’re doing great, it’s almost over.’” (Participant 19) (Sobel et al. 2018)  “’You were raped, you were molested.’ Don’t say raped because women, that triggers them and that hurts women.” (Participant 17) (Sobel et al. 2018) |
